# Evolving Role of Social Media in Health Promotion: Updated Responsibilities for Health Education Specialists

**DOI:** 10.3390/ijerph17041153

**Published:** 2020-02-12

**Authors:** Michael Stellefson, Samantha R. Paige, Beth H. Chaney, J. Don Chaney

**Affiliations:** 1 Department of Health Education and Promotion, East Carolina University, Greenville, NC 27858, USA; chaneye@ecu.edu (B.H.C.); chaneyj@ecu.edu (J.D.C.); 2 STEM Translational Communication Center, University of Florida, Gainesville, FL 32611, USA; paigesr190@ufl.edu

**Keywords:** social media, health education, health promotion

## Abstract

The use of social media in public health education has been increasing due to its ability to remove physical barriers that traditionally impede access to healthcare support and resources. As health promotion becomes more deeply rooted in Internet-based programming, health education specialists are tasked with becoming more competent in computer-mediated contexts that optimize both online and offline consumer health experiences. Generating a better understanding of the benefits and drawbacks to using social media in the field is important, since health education specialists continue to weigh its advantages against potential concerns and barriers to use. Accordingly, this Special Issue aims to explore social media as a translational health promotion tool by bridging principles of health education and health communication that examine (1) the method with which social media users access, negotiate, and create health information that is both actionable and impactful for diverse audiences; (2) strategies for overcoming challenges to using social media in health promotion; and (3) best practices for designing, implementing, and evaluating social media forums in public health. In this commentary, we discuss the updated communication and advocacy roles and responsibilities of health education specialists in the context of social media research and practice.

## 1. Introduction

Our understanding of health and the impact of behavioral, sociocultural, and system-level factors on health outcomes has evolved significantly over the past several decades [1]. Advances in technology are central to this evolution, as adoption of mobile devices connected to the Internet continues to grow across sociodemographic groups and geographic regions. One technological advancement accessed regularly is social media, which is used by 2.82 billion people worldwide [2]. Social media is defined as activities, practices, and behaviors among communities of users who gather online to share information, knowledge, and opinions using conversational media [3]. There are tens of thousands of health-promotion-related social media websites that are currently available to the public [1]. In health promotion, social media is commonly accessed for networking and community building purposes, as well as for informing healthcare decision-making between patients and providers [4].

The use of social media in public health education and promotion has been increasing in the United States (U.S.), due, in part, to its ability to remove physical barriers that traditionally impede access to healthcare support and resources. In 2017, Dr. Zsuzsanna Jakab, The World Health Organization (WHO)’s Regional Director of Europe, described the intersection of electronic health (eHealth) in public health as a “beautiful marriage“ that celebrates the global commitment and dedication towards reaping the benefits of eHealth for all [5]. Patients, clinicians, mobile health, and social media all play unique roles in health promotion, highlighting the need to for secure data management that can facilitate more personalized medicine and more equitable public health policies [5]. Today, it is difficult to imagine public health without social media. Although social media is viewed as acceptable and usable among multiple audiences and shows much promise in promoting health equity among disadvantaged populations (e.g., low income, rural, and older adults) [6], there remains inconsistent empirical evidence on the effectiveness of social media to improve public health outcomes and trends [7,8]. In order to optimize the potential of social media to improve public health, there is a need to effectively leverage these technological tools to create scalable, culturally adapted health promotion programs and campaigns. Unfortunately, evidence remains limited on how to do this within the field of health promotion [6,9]. Generating a better understanding regarding the benefits and drawbacks to using social media in health promotion is important, since health education specialists weigh its advantages against potential concerns over misinformation being shared to the public at large [10].

Central to social media is interactivity. Social media facilitates greater information sharing and opportunities for community building through an Internet-mediated dialogue that allows users to create their own content (e.g., blogs, online discussion boards). This content, in turn, can become invaluable for health education specialists who are seeking formative research to design, adapt, and evaluate programs and campaigns with priority audiences. Consistently, social media hosts opportunities for consumers to exchange strategic health messages on popular social media channels, including Facebook, YouTube, and Pinterest, through various modalities (e.g., text, image, video, and gif) [11]. Moreover, recent analytic advancements have strengthened the capacity of researchers and practitioners to compute and analyze metrics that evaluate the process of implementing social media, as well as any health-related impacts and outcomes associated with its implementation. As such, new collaborative evaluation methods are being deployed to improve the integration of social media within health-related interventions. While progress is being made, there remain significant challenges inhibiting the widespread acceptance, adoption, and use of social media in health promotion [4,12,13]. Further examining the impact of communication and advocacy within social-media-based interventions and campaigns is central to this endeavor.

Health education specialists play a critical role in creating, managing, and monitoring health promotion programs. As health promotion becomes more deeply rooted in Internet-based programming, health education specialists are tasked with becoming more competent in computer-mediated contexts that optimize both online and offline consumer health experiences. Accordingly, this Special Issue aims to explore social media as a translational health promotion tool by bridging principles of health education and health communication that examine: (1) the method with which social media users access, negotiate, and create health information that is both actionable and impactful for diverse audiences; (2) strategies for overcoming challenges to using social media in health promotion; and (3) best practices for designing, implementing, and evaluating social media campaigns and forums in public health. In this commentary, we discuss updated communication and advocacy roles and responsibilities of health education specialists in the context of using social media in research and practice.

## 2. Updated Social-Media-Related Roles and Responsibilities of Health Education Specialists 

The National Commission for Health Education Credentialing, Inc. (NCHEC) and the Society for Public Health Education (SOPHE) recently co-sponsored a new health education specialist practice analysis. A panel of 17 individuals with diverse backgrounds (i.e., work setting, experience level, education background, demographics, and geographic settings) that affect the practice of health education conducted a validation study, known as *Health Education Specialist Practice Analysis II (HESPA II 2020)* to re-verify the entry- and advanced-level responsibilities, competencies, and subcompetencies that provide the foundation for the professional preparation and development of all health education specialists [14]. A broad cross-section of both certified and noncertified health education specialists from all 50 U.S. states volunteered to participate in the study. Study participants were contacted via existing lists of the sponsoring organizations with additional assistance provided by the Coalition of National Health Education Organization (CNHEO) and national and state affiliates of major health education associations. Two online surveys, one focusing on competencies and one focusing on knowledge areas, were available for a three-month window from November 2018 to January 2019, resulting in 3,851 usable surveys [14].

Findings from this research provided significant implications for professional preparation, continuing education, and practice for the health education profession. Moreover, *HESPA II 2020* produced a new hierarchical model with eight areas of responsibility, 35 competencies, and 193 subcompetencies [13]. Within these new areas of responsibility, Advocacy (Area V) and Communication (Area VI) were designated as standalone areas of responsibility that contained a variety of new competencies and subcompetencies that reflected the increasing importance of using social media in the process and practice of health education. Table 1 outlines these two areas of responsibility with five associated health education specialist competencies and six subcompetencies that directly mention social media use.

### 2.1. Engage Coalitions and Stakeholders in Addressing Public Health Issues Using Social Media

Health education specialists are tasked with specifying strategies, timelines, and roles and responsibilities to address proposed policy, system, or environmental changes through social media. Social media allows for synchronous and asynchronous communication in a centralized, readily accessible digital location where a high degree of transparency exists. Social media can assist health education specialists in building a network of supporters, particularly for advocacy efforts [15]. These interactive, digital tools can be used to effectively expand the reach and inclusivity of advocacy campaigns to engage stakeholders to support public health issues, regardless of geographic location and timing [16]. Specifically, when used with traditional, relationship-building strategies, social media can bolster outreach approaches and reinforce relationships among stakeholders, including public health education coalition groups. This is done through promoting dialogue between leaders and supporters, as well as increasing collaborative communication among stakeholder groups. Additionally, social media tools are highly cost-effective for expanding communication among stakeholders and coalition groups interested in supporting public health education and promotion issue(s) [17,18]. Therefore, social media technologies have potential to improve communication among stakeholders in order to further engage supporters for successful social change. However, building relationships with stakeholders and coalitions through traditional communication channels, while supporting these relationships through the use of social media technologies, is ideal for fostering lasting and productive stakeholder relationships for addressing public health issues [18]. This allows for the opportunity to develop and nurture collaborative relationships among decision makers, which can include diverse stakeholders such as community members, organizations, and policymakers.

### 2.2. Engage in Health Policy Advocacy Through Leveraging Social Media

Social media has become a critical tool in advocating for health policy, including its development, planning, and reform. Engagement with advocates is a key element in advocating for health policy, and social media provides a platform for new supporters and the general public to become aware of the important issues [19]. In addition, social media tools create widespread access to public officials, many of whom have their own social media websites, for the opportunity to share information regarding health policy issues impacting constituents. While these technologies create the digital platform to increase awareness and evoke support for health policy advocacy, health education specialists must strive to promote actions that results in social change through advocacy efforts. Social media can complement traditional advocacy approaches to shift policy priorities for supporting health policy. In a framework developed by Scott and Maryman [18], social media and advocacy are aligned through empowerment and organization theories for shifting policy priorities. Specifically, the model suggests that quality social media presence must involve 1) critical awareness —engaging supporters through awareness of an issue that drives the desire to actively support the cause, 2) relationship building—creating relationships in a digital space and with face-to-face interactions that move passive supporters to active supporters, and 3) mobilizing action—creating action through both social media-supported online and offline forms of political engagement [18]. Successful social media campaigns for health policy advocacy require health education specialists to utilize planning and evaluation skills to effectively assess the use of social media in this capacity.

### 2.3. Determine Factors that Affect Health Communication on Social Media with the Identified Audience(s)

It is important for health education specialists to identify communication channels, such as social media, that are available to and used by their intended audience. Being digital-media-proficient means being able to meet priority populations where they are to bring about change within the physical, social, and online environments in which they live, work, and play. There are many challenges to effectively using digital media platforms, such as social media, within health education/promotion interventions and campaigns. These challenges are directly tied to the nature of social media itself, where health education specialists cannot fully control what, when, and how health information is shared. In some respects, social media can be considered the “wild west” for health information. Users can freely engage and interact with health information that may or may not be accurate or supported by empirical evidence. While challenges are to be expected, engagement can be maximized on social media through managing misinformation, reducing agency barriers to use, measuring audience reach and impact of posted messages/content, and keeping up with new trends in social media adoption and use. To effectively engage diverse audiences, there are several steps that can be followed to adopt a more strategic approach to social media use in health promotion: 1) understand how the priority population uses social media, 2) identify evidence-based social media strategies, 3) select appropriate communication times and channels, and 4) determine which types of social media apps will engage your audience most often in a meaningful way [20].

### 2.4. Deliver Health Message(s) Effectively Using Social Media

As reflected in *HESPA II 2020* competencies and subcompetencies, health education specialists are tasked with fine tuning their message delivery to ensure that intended audiences are being reached. This involves using current and emerging communication tools and digital media (e.g., social media management tools and platforms) to engage audiences. There are various social media tools, guidelines, and best practices that health education specialists can use for this purpose [20,21]. For example, health education specialists should stay abreast of new forms of social media that are accessed regularly (i.e., daily or almost daily) by intended users. Next, consider adopting a social media policy. A formal social media policy on relevant topics such as hashtag use, tagging, communicating, and updating content can limit destructive posts that adversely impact online communities [21]. Moreover, policy implementation facilitates productive interactivity that respects the diversity of user demographics, cultural backgrounds, and opinions. Finally, try to keep social media activity both lively and relevant. Skilled social media moderators are essential for maintaining social media pages and maximizing engagement through scheduling messages and responding promptly to user posts about current public health issues that are of concern. Moderators can provide invaluable social support that clinicians are often unable to offer, such as sharing insight about how to effectively communicate with healthcare providers [20].

### 2.5. Evaluate Health Promotion Activity Occurring on Social Media

Evaluation is a fundamental element of most all social media activities within the field of health promotion [20]. Process evaluation, or the measurement of factors that influence the success or failure of social media use (i.e., tracking social media analytics and performance indicators), is the most relevant type of evaluation to assess use of social media as part of an intervention or as a standalone tool [19]. Data from process evaluation enables key decision makers and other stakeholders to monitor program inputs (e.g., messages, videos, and chat sessions) and outputs (e.g., number of followers, number of likes, and number of comments left) of social media activity [11]. Tools such as social media analytics and data mining software can assist health education specialists in assessing the reach and dose of communication messages [22]. Analytics also help to extract useful patterns of user activity to measure the engagement, experience, and moderator responsiveness within online communities [23]. This type of social media data enables decision makers to learn from mistakes, make health promotion program modifications, monitor progress towards program goals, and justify the success of achieving desired health-related outcomes [20].

## 3. Conclusions

Social media provides an outlet to increase and promote translational health communication strategies and effective data dissemination, in ways that allow users to not only utilize but also create and share pertinent health information. Moreover, the use of social media for advocacy and communications in health promotion offers exciting new prospects for broader reach, greater efficiency, and lowered costs of communication and advocacy campaigns. As with other technological innovations in healthcare, these efficiencies may be viewed by those providing funding as an opportunity to decrease budgets and increase the scope of health promotion activity delivered by health education specialists and their organizations. This very may well result in a reduction in the use of more established communication channels (e.g., TV, radio, and print-based media) traditionally used for health promotion.

Although the application of social media in public health and health promotion has yielded some success in terms of generating support structures and networks for effective health behavior change, there are challenges and complications associated with social media use that also need to be addressed (e.g., managing misinformation, ensuring compliance with user privacy protections). While it is relatively straightforward to view social media use as a universal communication channel, especially for those who already use social media, the risk of using social media lies in reducing health information access among those who are not technologically ‘’connected’’. Social media is not likely to be an effective option for population subgroups include the elderly; the physically and cognitively disabled; and those with low text, technical, and eHealth literacy.

As health education specialists, we need to be wary of designing social media interventions or campaigns that are most suited to population segments that are comfortably well off, and text-, tech- and eHealth-literate. In addition, the use of social media by health education specialists faces significant headwinds from individuals or entities using social media to promote alternative views on health-related issues (e.g., anti-vaccinations, pro fad diets, and advocating for exclusionary healthcare policies). Some social media platforms have belatedly taken action to limit some of these discussions (e.g., Facebook with anti-vaccination groups), but the response is unlikely to be timely. We acknowledge that these types of completing voices are usually far better resourced than health education specialists who have limited resources to support robust social-media-based advertising campaigns. Therefore, we must be vigilant in monitoring and evaluating public health advocacy and communication that occurs on various popular social media websites.

Our Special Issue begins to tackle these important issues by bringing together international, multidisciplinary scholars who employed innovative methodologies to better understand how social media is used by multiple audiences for the purposes of health promotion and engagement. Specifically, these articles delve into the sociocognitive and affective factors that mediate the relationship between social media use, community engagement, and positive health outcomes. This was achieved by augmenting our understanding of traditional health education approaches with theories rooted in the complementary yet distinct disciplines of health communication. We sincerely hope that the new empirical knowledge generated within this Special Issue will help academic health education specialists, as well as other public health professionals, use more pragmatic paradigms for planning, implementing, and evaluating social media interventions and campaigns in the field of health promotion.

## Figures and Tables

**Table 1:** HESPA II 2020 competencies and subcompetencies that address health education specialist use of social media.

Area of Responsibility	Competency	SubCompetency
Advocacy	Engage coalitions and stakeholders in addressing health issues and planning advocacy efforts	Specify strategies, a timeline, and roles and responsibilities to address the proposed policy, system, or environmental change (e.g., develop ongoing relationships with decision makers and stakeholders, use social media, register others to vote, and seek political appointment)
	Engage in advocacy	Use media to conduct advocacy (e.g., social media, press releases, public service announcements, and op-eds)
Communications	Determine factors that affect communication with the identified audience(s)	Identify communication channels (e.g., social media and mass media) available to and used by the audience(s)
	Deliver the message(s) effectively using the identified media and strategies	Use current and emerging communication tools and trends (e.g., social media, community presentations, annual reports, and patient newsletters)
		Use digital media to engage audience(s) (e.g., social media management tools and platforms)
	Evaluate communication	Assess reach and dose of communication using tools (e.g., paper-based surveys, focus groups, semistructured in-depth interviews, data mining software, social media analytics, and website analytics) ^1^

^1^ Advanced 1 subcompetency not included in the entry-level, Certified Health Education Specialist (CHES^®^) examination. Subcompetency will be included in the Master Certified Health Education Specialist (MCHES^®^) examination.
